# Soybean image dataset for classification

**DOI:** 10.1016/j.dib.2023.109300

**Published:** 2023-06-07

**Authors:** Wei Lin, Youhao Fu, Peiquan Xu, Shuo Liu, Daoyi Ma, Zitian Jiang, Siyang Zang, Heyang Yao, Qin Su

**Affiliations:** a Nanjing Agricultural University, Nanjing, China; b Jiangsu University of Science and Technology, Zhenjiang, China; c Tonghua Normal University, Tonghua, China; d Jiangsu University of Technology, Changzhou, China

**Keywords:** Image processing, Image datasets, Soybean, Convolutional neural networks

## Abstract

This paper presents a dataset with ***5513*** images of individual soybean seeds, which encompass ***five categories***: **(Ⅰ) Intact, (Ⅱ) Immature, (Ⅲ) Skin-damaged, (Ⅳ) Spotted, and (Ⅴ) Broken**. Furthermore, **there are over 1000 images of soybean seeds in each category.** Those images of individual soybeans were classified into five categories based on the Standard of Soybean Classification (***GB1352-2009***) [1]. The soybean images with the seeds in physical touch were captured by an industrial camera. Subsequently, individual soybean images (227×227 pixels) were divided from the soybean images (3072×2048 pixels) using an image-processing algorithm with a segmentation accuracy of over 98%. The dataset can serve to study the classification or quality assessment of soybean seeds.

**Specifications Table**

**Table:** 

Subject	Computer Science, Agricultural Science
Specific subject area	Image processing, crop classification
Type of data	RGB images (24-bit, BMP format)
How data were acquired	The individual soybean images (227×227 pixels) were divided from the soybean images (3072×2048 pixels) via an image-processing algorithm.
Data format	24-bit RGB Raw Processed
Description for data collection	An image acquisition system (Fig. 2) was used to capture the soybean images (3072×2048 pixels) where the seeds are in physical touch. Then, an image-processing algorithm was adopted to split the individual soybean images (227×227 pixels) from the soybean images (3072×2048 pixels). Finally, the individual soybean images were saved in JPG format.
Data source location	Nanjing Agricultural University, Nanjing, China
Data accessibility	Repository name: Soybean Seeds Data identification number: https://doi.org/10.17632/v6vzvfszj6.6 Direct URL to data: https://data.mendeley.com/datasets/v6vzvfszj6 Instructions for accessing these data: Download the data from Soybean Seeds repository in ZIP formats.

## Value of the Data

• The soybean image dataset can meet the practical requirement of assessing soybean quality. Because those individual soybean images in our dataset were classified based on the Standard of Soybean Classification (***GB1352-2009***) [1].
• This dataset can complement other soybean seed image datasets, providing more available images of soybean seeds to develop better models.
• Researchers in soybean breeding may use this dataset and benefit.

## 1 Objective

There are no published datasets for studies on seed classification [2], [3], [4], [5], [6], [7], [8], [9]. Meanwhile, creating a dataset is laborious and time-consuming. In addition, non-public datasets could not validate algorithms and promote the development of seed classification.

Currently, researchers have yet to publish soybean seed image datasets, nor have they classified soybean seeds according to a common standard [2,4,[7], [8], [9]]. Therefore, we aim to construct a public dataset of individual soybean seed images based on the Standard of Soybean Classification (***GB1352-2009***) [1] for researchers studying the classification or quality assessment of soybean seeds.

## 2 Data Description

**Fig. 1:**
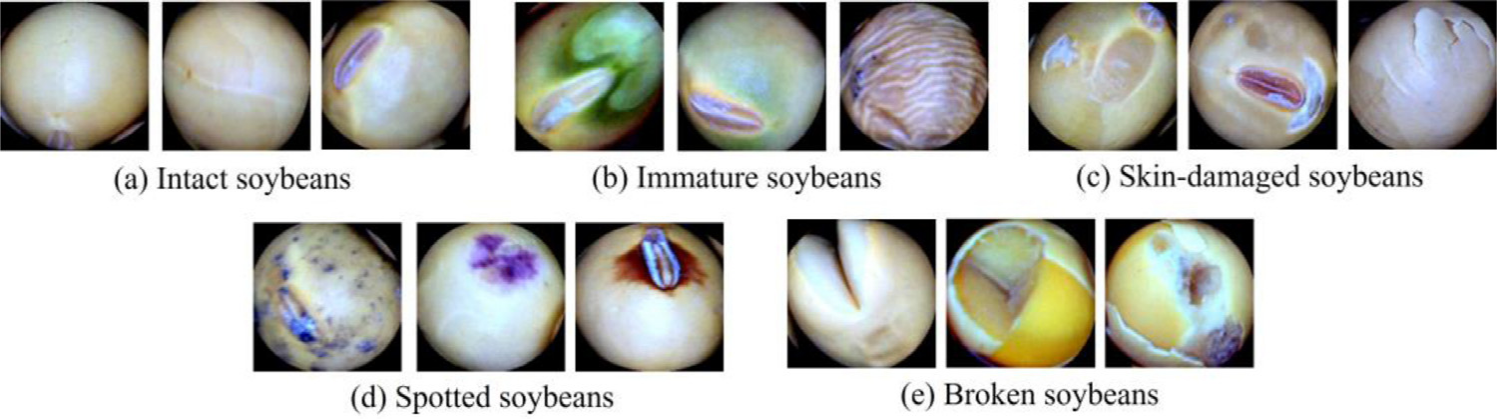
Picked soybean seed samples.

The image dataset of soybean seeds can serve to study the classification or quality assessment of soybean seeds. The dataset includes five-type of individual soybean seed images: **intact, spotted, immature, broken**, and **skin-damaged**, as shown in Fig. 1.

The individual soybean images (227×227 pixels) were generated from the soybean images (3072×2048 pixels) via an image-processing algorithm. Subsequently, the five types of individual soybean images were sorted according to the Standard of Soybean Classification (***GB1352-2009***) [1]. The following is a complete description of the soybean classification:

(1) Intact soybeans: complete and shiny soybeans.
(2) Immature soybeans: shrunken soybeans or soybeans with green parts.
(3) Skin-damaged soybeans: soybeans with damaged seed skin.
(4) Spotted soybeans: soybeans with disease spots on the surface.
(5) Broken soybeans: insect-bitten, split, or the soybeans are broken up to one-fourth of the volume of the seeds or larger.

The dataset contains a total of 5513 individual soybean seed images. Meanwhile, there are over 1000 images of individual soybean seeds in each category, as shown in Table 1.

**Table 1:** The description of the soybean seed dataset.

Folder	Account of images
Broken soybeans	1002
Spotted soybeans	1058
Immature soybeans	1125
Intact soybeans	1201
Skin-damaged soybeans	1127
Total number of images	5513

## 3 Experimental Design, Materials and Methods

### 3.1 Image Acquisition System

**Fig. 2:**
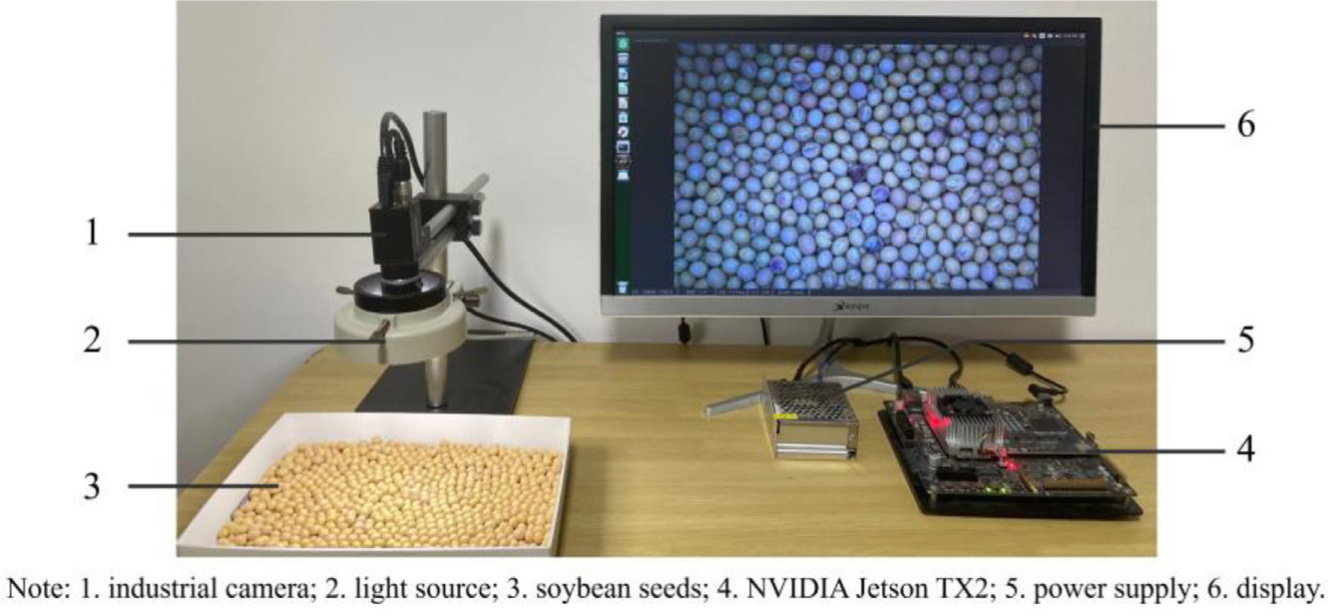
Image acquisition system.

The image acquisition system consists of an industrial camera (MV-CA060-11GM, HIKVISION Co., Ltd., Hangzhou, China), light source, NVIDIA Jetson TX2, power supply, and display, as shown in Fig. 2.

The light source was positioned parallel to the soybean seeds at approximately 135*mm*, providing a light intensity of around 2×10^3^*Lux*. The industrial camera was then placed parallel in the center of the light source about 143*mm* from the plane of soybeans. The industrial camera was connected to the NVIDIA Jetson TX2 via GigE. The exposure time of the industrial camera was set to 2×10^3^*us*. The soybean images (3072×2048 pixels) were saved in JPG format.

### 3.2 Image Processing

The image-processing algorithm based on [10] was constructed with C++ and Opencv Library (Version 3.4.8).

**Fig. 3:**
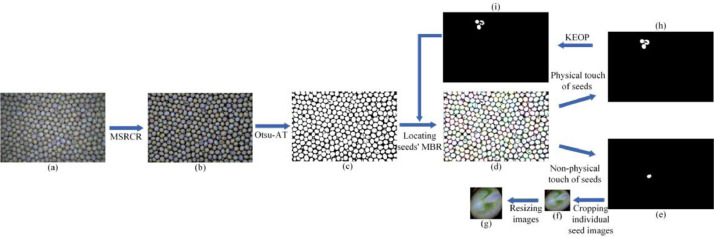
The flowchart of image processing: a) original image, b) enhanced image, c) binary image, d) locating individual seeds using MBR, e) binary image after mask with non-physical contact seeds, f) individual soybean image cropped out from the enhanced image, g) individual soybean image after resizing, h) binary image after mask with physical contact seeds, and i) binary image after mask that some tiny contact between soybean seeds were eliminated by KEOP.

In the algorithm, the Multi-scale Retinex with Color Restoration (MSRCR) [11] was employed to enhance the contrast of the soybean image. Otsu [12] adaptive thresholding (Otsu-AT) was applied to segment the foreground and background of the enhanced image. The minimum bounding rectangle (MBR) was used to locate those individual seeds on the binary image. Soybeans were masked according to the location of the MBR. The size of MBR was used to judge whether seeds were in physical contact. If the seeds were non-physically touching, those individual seed images were cropped out from the enhanced image and then resized as 227×227 pixels. If the seeds were physically touching, the erosion operation with the 13×13 kernel (KEOP) was applied to eliminate some tiny contact between seeds on the binary images after masking. Then, those seeds were relocated by MBR. Finally, those individual seed images were cropped out and then resized. The flowchart of image processing shows in Fig. 3.

### 3.3 Evaluation Metric

For evaluating the segmentation performance of the algorithm of the image processing, accuracy (Acc) was used as evaluation metric in this task.

(1) $$Acc=\frac{Number\;of\;images\;of\;properly\;segmented\;individual\;seeds}{Total\;number\;of\;images\;of\;segmented\;individual\;seeds}$$

### 3.4 Image Processing Experiments

The image-processing method that does not process those seeds in contact with each other achieves about 95.31% segmentation accuracy. However, we found that part of tiny touch seeds in soybean images (Fig. 3h) could be further segmented.

Table 2 shows that Erosion Operation (EOP) and Watershed Algorithm (WA) were used to address those seeds in tiny contact. The image-processing algorithm with the 13×13 kernel erosion operation had excellent segmentation accuracy (about 98.51%), with the average segment time for a seed approximately 103*ms*. Although the image-processing algorithm with the watershed algorithm can reach 98.60% segmentation accuracy, its average segment time for individual seeds is approximately 161*ms* which is over 1.5 times that of the image-processing algorithm with the 13×13 kernel erosion operation.

Our image-processing algorithm needs to achieve fast segmentation of soybean seeds and can be applied on resource-limited devices. We considered that the algorithm complexity of the watershed algorithm is considerably higher than that of the erosion operation, and erosion operation is more accessible to implement than the watershed algorithm. Furthermore, their segmentation accuracies are almost the same. Therefore, the image processing algorithm with the 13×13 kernel erosion operation was considered to split individual seed images from the soybean images.

**Table 2:** Different methods of eliminating some tiny contact between seeds.

Methods	Kernel size	Acc	Average segmentation time of a seed/*ms*
EOP	3×3	97.15%	98.64
	5×5	97.37%	98.98
	7×7	97.98%	99.86
	9×9	98.19%	101.02
	**13**×**13**	**98.51%**	**103.63**
WA	-	98.60%	160.68

### 3.5 Image Processing Conclusion

The image processing method with the 13×13 kernel erosion operation can achieve over 98% segmentation accuracy for the images of soybeans in physical contact. And it takes approximately 103*ms* to segment individual soybean seeds on NVIDIA Jetson TX2, which may meet the requirement of online segmentation of touching soybean seed images.

## Ethics Statements

This paper is the authors’ own original work, which has not been previously published elsewhere. The authors declare compliance with the publication code of ethics of this journal.

## CRediT authorship contribution statement

**Wei Lin:** Writing – original draft, Writing – review & editing. **Youhao Fu:** Writing – review & editing. **Peiquan Xu:** Writing – review & editing. **Shuo Liu:** Writing – review & editing. **Daoyi Ma:** Writing – review & editing. **Zitian Jiang:** Writing – review & editing. **Siyang Zang:** Writing – review & editing. **Heyang Yao:** Writing – review & editing. **Qin Su:** Writing – review & editing.

## Declaration of Competing Interest

The authors have no conflicts of interest.

## Data Availability

Soybean Seeds (Original data) (Mendeley Data).
